# CC and CXC chemokines play key roles in the development of polyomaviruses related pathological conditions

**DOI:** 10.1186/s12985-021-01582-4

**Published:** 2021-06-03

**Authors:** Mohammad Hassan Mohammadi, Ashraf Kariminik

**Affiliations:** 1 Department of Pediatrics, Faculty of Medicine, Zabol University of Medical Sciences, Zabol, Iran; 2 Department of Microbiology, Kerman Branch, Islamic Azad University, Kerman, Iran

**Keywords:** Polyomaviruses, CXC chemokines, CC chemokines, Nephropathy, Cancer

## Abstract

It has been reported that polyomaviruses are the microbes which can be a cause of several human pathological conditions including cancers, nephropathy, progressive multifocal leukoencephalopathy and gynaecological disease. Although investigators proposed some mechanisms used by the viruses to induce the disorders, the roles played by chemokines in the pathogenesis of polyomaviruses infections are yet to be clarified. This review article investigated recent studies regarding the roles played by chemokines in the pathogenesis of the polyomaviruses infections. The research in the literature revealed that CXC chemokines, including CXCL1, CXCL5, CXCL8, CXCL9, CXCL10, CXCL11, CXCL12 and CXCL16, significantly participate in the pathogenesis of polyomaviruses. CC chemokines, such as CCL2, CCL5 and CCL20 also participate in the induction of the pathological conditions. Therefore, it appears that CXC chemokines may be considered as the strategic factors involved in the pathogenesis of polyomaviruses.

## Introduction

It has been demonstrated that human polyomaviruses are the viruses responsible for inducing some pathologic conditions and malignancies in human [1, 2]. However, both the JC and the BK viruses are potential risk factor for human disorders. For example, BK virus is the main cause of hemorrhagic cystitis in recipients of bone marrow transplantation, while JC virus can induce progressive multifocal leukoencephalopathy in immunocompromised patients [3]. Additionally, transplant nephropathy is another disease which is induced by BK virus infection, and is recognized as a main cause of renal allograft failure [3]. Moreover, it has been reported that various neoplastic disorders and autoimmune conditions are associated with infection with BK virus [3]. Due to the fact that the JC and BK virus proteins can interact with a number of cellular target proteins in the central nervous system, they can dysregulate pathways involved in the cell cycle, DNA repair, and others, which are the main altered molecules during cancers [4]. Therefore, the viruses, especially JC virus, are considered as the potential inducers of brain tumors.

The main mechanisms used by the viruses to induce the disorders are yet to be clarified completely. It has been hypothesized that immune system may participate in the transformation of the target tissues [5]. Chemokines are the main molecules secreted by the immune and non-immune cells and are crucial for development of tumors and other complications [6]. This review article addresses the main mechanisms played by chemokines in the pathogenesis of polyomaviruses-related pathological conditions.

## Introduction of polyomaviruses

The human polyomaviruses are the 40–45 nm viruses which contain the spherical nonenveloped capsids and also the 5000 base pairs double-stranded DNA genomes [1, 2]. Human polyomaviruses have a genome which is divided into two important regions called, the early and late regions [7]. Accordingly, the early and late regions code large T-antigen and small t-antigen, capsid proteins (VP)-1, VP-2, and VP-3, respectively [8]. Additionally, the late gene region in two important members of the polyomaviruses, BK and John Cunningham (JC) polyomaviruses, also code another protein, agnoprotein, which participates in the assembly of the viral capsid and also the virion release from the infected cells [9, 10].

As mentioned, BK virus is an important member of the polyomaviruses whose infection and activation are associated with various human clinical pathogenesis [5]. JC virus is another member of the polyomaviruses and was discovered in a patient who suffered from Hodgkin’s lymphoma and progressive multifocal leukoencephalopathy (PML) [11]. Recent genomic sequencing technologies revealed 11 additional polyomaviruses such as human polyomavirus 3 (KI virus), 4 (WU virus), 5 (Merkel cell polyomavirus), 6, 7, 8, 9, 10 (Malawi or MW polyomavirus), 11 (Saint Louis polyomavirus), 12 and 13 (New Jersey polyomavirus) [11].

Although all of the polyomaviruses participate in the human disease pathogenesis, BK and JC viruses are the most important polyomaviruses regarding their related disease severities and also their prevalence among the human population [12–14]. The prevalence of BK and JC viruses is variable based on the age and location. However, epidemiological investigations, which were performed based on the seropositive reactions against BK and JC viruses, revealed that 65–90% of adults have antibody against the viruses [15].

Accordingly, latent form of BK virus is prevalent in several ethnic populations [16]. Following Immunosuppression and also interactions of the cellular proteins with the large T antigen, BK virus is re-activated and induces the polyomavirus related complications such as nephropathy [16, 17]. Due to the sequences of the transcriptional control regions, BK virus is divided to two different forms, archetype and rearranged variants [18]. Archetype can be separated from urine in both latent and activated forms and also is the transmissible form, while rearranged variants present in the serum/plasma samples of BK reactivated patients [19, 20]. BK virus reactivation is a main cause of nephropathy and hemorrhagic cystitis in the infected patients [12–14, 21]. Four genotypes, genotypes I (the most prevalent genotype), II, III, and IV, have been reported for the BK virus, which are defined based on the sequence variations in VP1 [22, 23], and are corresponded to the BK virus serotypes, BK, SB, As, and IV, respectively [23], while, JC virus has one VP-1 serotype and 12 subtypes [24]. For more information regarding the genotypes of BK and JC viruses, please read the articles by Hirsch et al. [25] and Yogo et al. [26].

Polyomaviruses, including BK and JC viruses are transmitted via the respiratory route (the common route), blood transfusion, fecal/urine oral, and transplantation [27]. The polyomavirus infections may disseminate to the other tissues, such as urinary tract and immune cells, then replicate in the tissue cells [28, 29]. It has been reported that JC Virus can be associated with PML and nephropathy, while several investigations proved the roles played by BK viruses in the pathogenesis of nephropathy, hemorrhagic cystitis and ureteric strictures [26, 30]. Additionally, the crucial roles played by polyomavirus middle T-antigen (MMTV-PyMT) in induction of cancers, such as breast cancer, have been documented previously [31].

## Chemokines

Chemokines are small chemotactic cytokines with 8–12 kDa, which share four cysteines to make the chemokine fold. The directions of the cysteines are used in the nomenclature of the chemokines. Accordingly, the chemokines are divided into four groups, including CC, CXC CX3C and XC chemokines [32]. The chemokines perform their functions via interactions with their corresponded receptors (Chemokine receptors), hence, they are called as "Ligand", hence, the CC, CXC CX3C and XC chemokines are called CCLs, CXCLs, CX3CL1 (this group has one member) and XCls [33]. The CC chemokines, which are known as β-chemokines, consist of 27 distinct members. Although there are CCL1 to CCL28, it has been reported that CCL9 and CCL10 are the same chemokines [33]. There are 17 different CXC chemokines, which are known as α-chemokines, in the mammalian. The CXC chemokines are divided into two main categories, ELR (glutamic acid-leucine-arginine) positive and negative, due to the existence or lack of the specific motif before the first cysteine [34]. The ELR positive chemokines, such as CXCL8, are the main chemotactic factors for neutrophils, via interactions with CXCR1 and CXCR2, while the ELR negative chemokines, such as CXCL13, play key roles in the attraction of lymphocytes [35]. XCL1 (lymphotactin-α) and XCL2 (lymphotactin-β), which are known as γ chemokines, are two members of the XC chemokines and participate in the dendritic-cell-mediated cytotoxic immune response and attractions of the CD8^+^ and CD4^+^ T cells, respectively [36]. CX3C chemokines, which are also known as δ-chemokines, have one member entitled CX3CL1 or fractalkine, and play two distinct roles as chemoattractant and as an adhesion molecule [37].

**Fig. 1:**
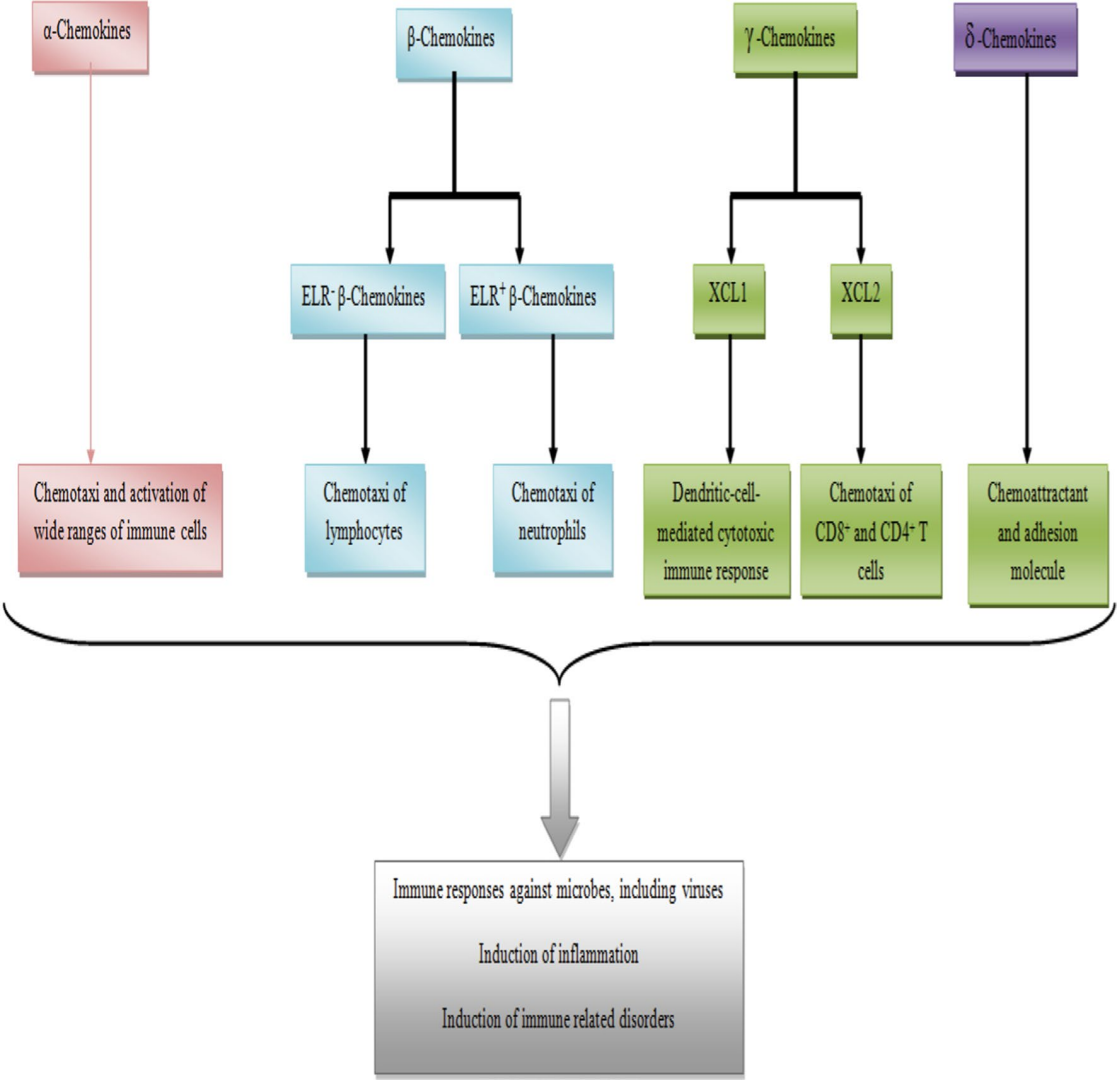
Chemokine family and their functions. The figure illustrates that chemokines are divided to four members and some of them interact with a range of immune cells. Finally chemokines participate in the induction of immune responses against microbes, including viruses, and in some cases induce immune related disorders

The primary functions of most chemokines are induction of inflammation, especially after viral infections. However, they play key roles in other immune response functions, such as angiogenesis/angiostasis and development of tissues [6]. Figure 1 illustrates the chemokine family and their functions. Thus, next sections of this project describe the plausible critical roles played by chemokines during polyomavirus infections.

## Chemokines and polyomaviruses

The searching in the database centers, including Google scholar, Pubmed and Scopus, revealed that there were no investigations regarding the relationship between polyomaviruses and CX3C and XC chemokines. However, several investigations evaluated the roles played by CC and CXC chemokines during exposure to polyomaviruses. Accordingly, several investigations demonstrated that polyomaviruses are the main causes of chemokines up-regulation. For example, Marzocchetti et al., reported that JC virus is a main inducer of CCL2 expression in the cerebrospinal fluid of the infected patients [38]. A study by De-Simone et al., demonstrated that, although JC virus infection leads to up-regulation of CCL5, it reduced expression of CXCL9 and CCL2 chemokines in the peripheral blood mononuclear cells (PBMCs) [39]. Another study by Cason and colleagues showed that CCL5 and CXCL9, the important factors that participate in the induction of cell differentiation, transformation, and chronic inflammation in the mesenchymal stromal cells, are up-regulated following infection with polyomaviruses such as JC virus [40]. Previous investigations revealed that CCL5 plays potential roles against viral infections via recruitment of T cells and other leukocytes into infected sites and also induction of either proliferation or activation of natural killer (NK) cells, the most important innate immune cell against viral infected cells [41]. Our previous investigations on the patients with kidney transplantation revealed that BK virus induces kidney nephropathy in the patients via up-regulation of CXCL9 [42], CXCL10 [43], and CXCL11 [44]. Increased expression of CXCL10 by BK virus infected endothelial cells have also been reported by An and colleagues [45]. However, BK virus regulates expression of CXCL9 and CXCL11 at both transcription and translation levels, while it regulates CXCL10 at translation levels only [42–44]. Schachtner et al. [46] and Jackson et al. [47] also confirmed the results and reported that BK virus replication has a positive correlation with up-regulation of CXCL9 and CXCL10. Due to the fact that CXCL9 and CXCL10 significantly increased recruitment of cytotoxic lymphocytes (CTLs), NK cells, NK-T cells and macrophages [48, 49], hence, the chemokines play key roles in suppression of viral infections, including polyomaviruses. Therefore, it seems that polyomaviruses play key roles in the induction of expression of the chemokines and in some cases, they regulate the expression of the molecules to overcome immune responses and also induction of some tumor associated functions such as angiogenesis. However, it has been demonstrated that, alteration in expression of the chemokines by polyomaviruses is related to interactions of the viruses by the innate immunity receptors such as toll like receptors (TLRs) and intracellular sensors such as retinoic acid inducible gene-I (RIG-I) [50]. In other words, interactions of polyomaviruses with innate immune receptors lead to regulation of expression of the chemokines, which may make activation of normal immune responses to the viruses and eradication/suppression of them possible. Several investigations proved the roles played by chemokines against viral infections to overcome the microbes [51–53], thus, it is plausible that chemokines play key roles against polyomaviruses too. Above investigations also proved the roles played by chemokines against polyomaviruses. However, there are some documents which demonstrated that chemokines are unable to inhibit JC virus replication and also its gene expression in the glial and primary human astrocytes cells [39]. Non-interestingly chemokines in association with other immune responses are the most common weapon against polyomaviruses and are the main mechanisms for suppression of the viruses in the immune-compatible individuals.

Chemokines also may reduce polyomaviruses-related pathological functions. For example, Gao et al., reported that CCL5, via interaction with CCR5, significantly participates in the reduction of polyomavirus middle T-antigen (MMTV-PyMT) related mouse primary breast cancer through the attraction of immune cells, decreases expression of glucose transporter-1 (GLUT-1) cell surface, and subsequently decreases glucose uptake and also the intracellular AATP and lactate levels [31].

The roles of the chemokines in the pathogenesis of polyomaviruses are the main research fields of the investigators. Thus, recent studies explore the negative roles played by chemokines during polyomaviruses infections. A study by Comar et al., showed that polyomaviruses deteriorate the pathogenesis of Malignant Mesothelioma via induction of CCL5 expression in the in vivo condition [54]. Steiner and colleagues demonstrated that using bindarit, as a CCL2 synthesis inhibitor, leads to suppression of polyomavirus-related breast cancer development by down-regulation of CCL2 in the tumor tissue, but not in the plasma [55]. Interestingly, a study in the Merkel cell polyomavirus (MCV) showed that polyomavirus large T antigen, plays positive roles in up-regulation of CXCL12 and its related receptors, including CXCR3 and CXCR4 [56]. Another study demonstrated that polyomaviruses induces expression of CCL2 and CXCL12 to recruit tumor associated macrophages to the polyomavirus-related breast cancer tissue [57]. Tumor associated macrophages are the important cells to increase tumor progression and metastasis [58]. Additionally, it has been demonstrated that CCL2 and CXCL12 are the potential chemokines to induce angiogenesis, the main mechanisms that lead to tumors development [33]. Boyle and colleagues showed that CCR6, the chemokine receptor for CCL20, through recruitment of tumor associated macrophages, plays key roles in the induction of polyomavirus-related mammary neoplasia [59]. The roles played by the polyomavirus large and small T antigens in the induction of expression of CXCL8 in the MCV have been documented by Richards and colleagues [60]. The investigators demonstrated that expression of the pro-inflammatory chemokine is a main cause of malignancy in the cell line [60]. Thus, it appears that chemokines are the important molecules which are used by polyomaviruses to develop polyomaviruses-related cancers through an increase in angiogenesis and recruitment of some immunosuppressor cells to the tumor tissues.

Additionally, it has been documented that polyomaviruses are the main causes of nephropathy. Ribeiro et al., reported that BK virus induces transplantation associated nephropathy by up-regulation of CCL5 and CXCL8 in tumor necrosis factor (TNF)α/TNF receptor system dependent manner [61]. The positive correlation between expression of intragraft tubulointerstitial cell CCL2 and polyomavirus replication can be considered as a risk for induction of nephropathy in the transplanted kidney [62]. A study by Ho et al., revealed that up-regulation of CXCL10 in the urine of the patients who suffered from renal allograft inflammation has increased tissue BK virus load [63]. Our previous investigations also proved the roles played by CXCL9, CXCL10 and CXCL11 in the induction of nephropathy in the BK virus infected patients who underwent kidney transplantation [42–44]. CXCL10 is a molecule that suppresses angiogenesis [49] and, hence, it may be concluded that the molecule might increase the nephropathy risk via either suppression of angiogenesis and tissue repair or elevation of inflammation. Therefore, chemokines are the important molecules in induction of polyomaviruses-related nephropathy too. As mentioned previously, JC virus is the main cause of PML in the human [26, 30]. Due to the closed relation between polyomaviruses and chemokines, it is plausible that chemokines may participate in the pathogenesis of PML. Darbinyan and colleagues reported that JC virus suppresses differentiation of oligodendrocyte progenitor cells, a main mechanism which leads to PML, via dysregulation of CCL5, CXCL1, CXCL5, CXCL8, CXCL10 and CXCL16 [64]. Another investigation proved the roles played by JC viruses and revealed that JC virus agnoprotein is the main molecule responsible for reduction of CXCL5, and consequently, activation of apoptotic signaling pathways in the neurons, the main mechanism for induction of PML [65]. Thus, it may be concluded that chemokines, especially CXC chemokines play significant roles in the pathogenesis of PML. However, it needs to be explored by further studies via investigations of other CC chemokines and also CX3C and XC chemokines. Additionally, the roles played by polyomaviruses in the induction of gynaecological disease in CXCL8 dependent manner have also been demonstrated by Zhang and colleagues [66]. A complementary investigation showed that the polyomavirus large T antigen is the molecule responsible for up-regulation of CXCL8 [67]. Based on the fact that CXCL8 is the main chemotactic factor for neutrophils [33], it may be hypothesized that polyomaviruses induce gynaecological diseases by increasing inflammation in neutrophil dependent manner.

The main mechanisms which lead to altered expression of the chemokines during polyomaviruses infections in the human are yet to be clarified. It has been hypothesized that genetic variations within the chemokine genes can be associated with alteration in expression of the chemokines [68]. Multiple investigations regarding the genetic variations within chemokine genes and their relations with polyomaviruses related diseases do not exist. However, a genetic study by Guerini and colleagues revealed that there is an association between CCL5, but not CCR5, CCR2, CXCL12, single nucleotide polymorphism (SNP) and severity of JC virus-related PML [69]. Thus, it appears that more investigations need to be performed to clear the roles played by genetic variations in the outcome of polyomaviruses infections and their related pathogenesis.

## Conclusion

**Fig. 2:**
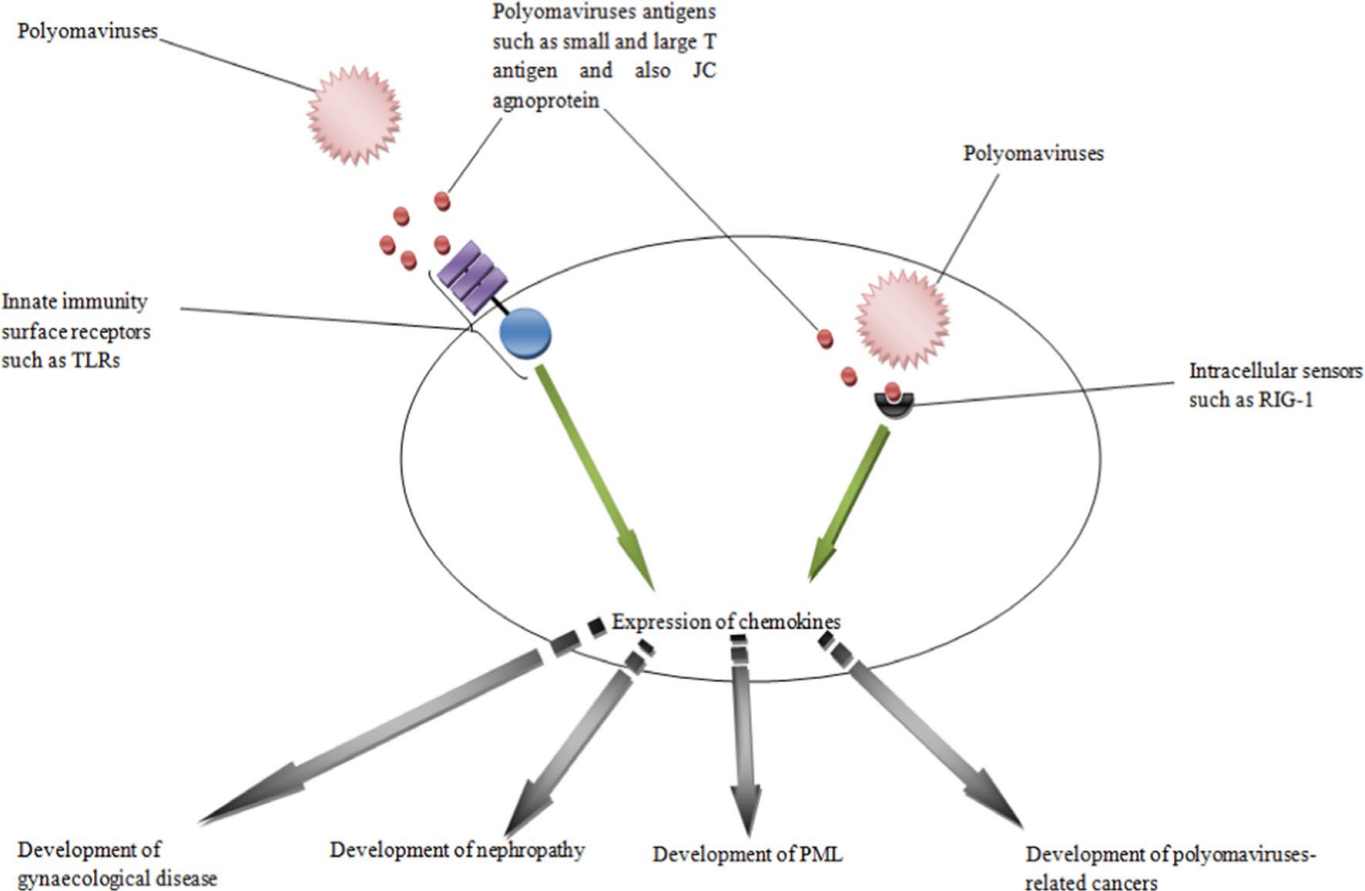
Pathological roles played by chemokines during polyomaviruses infections. The figure shows that polyomaviruses and their related antigens induce expression of chemokines, which leads to development of polyomaviruses-related cancers, PML, nephropathy and gynaecological disease

Collectively, chemokines play significant roles against polyomaviruses and suppress them, however, they are the critical responsible molecules that participate in the polyomaviruses related pathogenesis, including development of tumors, nephropathy, gynaecological disease and PML. Figure 2 illustrates the main physiological and pathological roles played by chemokines during polyomaviruses infections. Due to the results it may also be concluded that CXC chemokines, including CXCL1, CXCL5, CXCL8, CXCL9, CXCL10, CXCL11, CXCL12 and CXCL16, significantly participate in the pathogenesis of polyomaviruses. CC chemokines, such as CCL2, CCL5 and CCL20 also participate in the induction of pathological conditions. Therefore, it can be hypothesized that future investigations using specific antagonists for chemokines, for instance using antagonist for CXCL10 in nephropathy, can open an avenue to development of molecular therapy of polyomaviruses related disorders. Additionally, immunotherapy against some specific chemokines such as CCL2 may alter the polyomaviruses-related tumor microenvironment, which needs to be explored by future studies.

## Data Availability

Data and materials are available.

## Abbreviations

PML: Progressive multifocal leukoencephalopathy
CXCL: CXC ligand
VP: Capsid proteins
JC: John Cunningham
ELR: Glutamic acid-leucine-arginine
PBMCs: Peripheral blood mononuclear cells
TLR: Toll like receptor
RIG-I: Retinoic acid inducible gene-I
MMTV-PyMT: Polyomavirus middle T-antigen
GLUT-1: Glucose transporter-1
MCV: Merkel cell polyomavirus
TNF: Tumor necrosis factor
SNP: Single nucleotide polymorphism
